# 1-[3-(4-Chloro­phen­yl)isoquinolin-1-yl]-3,5-diethyl-1*H*-pyrazole

**DOI:** 10.1107/S1600536809055731

**Published:** 2010-01-16

**Authors:** F. Nawaz Khan, P. Manivel, K. Prabakaran, Venkatesha R. Hathwar, Seik Weng Ng

**Affiliations:** a Chemistry Division, School of Science and Humanities, VIT University, Vellore 632 014, Tamil Nadu, India; b Chemistry Division, School of Advanced Sciences, VIT University, Vellore 632 014, Tamil Nadu, India; c Solid State and Structural Chemistry Unit, Indian Institute of Science, Bangalore 560 012, Karnataka, India; d Department of Chemistry, University of Malaya, 50603 Kuala Lumpur, Malaysia

## Abstract

The title compound, C_22_H_20_ClN_3_, is composed of a dialkyl-substituted pyrazole ring connected to an aryl-substituted isoquinoline ring system with a dihedral angle of 55.8 (1)° between the pyrazole ring and and the isoquinoline ring system. The dihedral angle between the chloro­phenyl ring and the isoquinoline ring system is 28.3 (1)°.

## Related literature

For medicinal applications of hydrazine derivatives, see: Broadhurst *et al.* (2001 ▶).
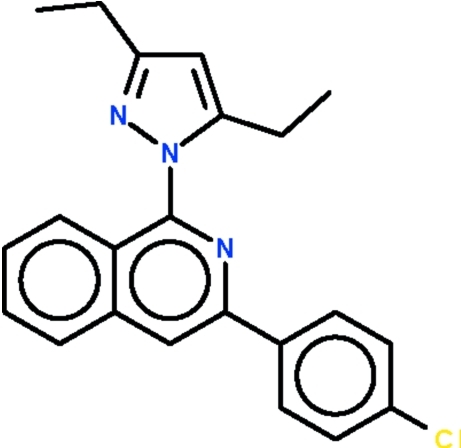


## Experimental

### 

#### Crystal data

C_22_H_20_ClN_3_
*M* *_r_* = 361.86
Monoclinic, 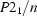

*a* = 8.4484 (6) Å
*b* = 15.0386 (12) Å
*c* = 15.4894 (11) Å
β = 96.763 (1)°
*V* = 1954.3 (3) Å^3^
*Z* = 4
Mo *K*α radiation
μ = 0.21 mm^−1^
*T* = 290 K
0.25 × 0.18 × 0.15 mm

#### Data collection

Bruker SMART area-detector diffractometer
Absorption correction: multi-scan (*SADABS*; Sheldrick, 1996 ▶) *T*_min_ = 0.951, *T*_max_ = 0.970
14613 measured reflections
3703 independent reflections
2235 reflections with *I* > 2σ(*I*)
*R*_int_ = 0.040

#### Refinement

*R*[*F* ^2^ > 2σ(*F* ^2^)] = 0.062
*wR*(*F* ^2^) = 0.177
*S* = 1.03
3703 reflections
235 parameters
H-atom parameters constrained
Δρ_max_ = 0.46 e Å^−3^
Δρ_min_ = −0.19 e Å^−3^

### 

Data collection: *SMART* (Bruker, 2004 ▶); cell refinement: *SAINT* (Bruker, 2004 ▶); data reduction: *SAINT*; program(s) used to solve structure: *SHELXS97* (Sheldrick, 2008 ▶); program(s) used to refine structure: *SHELXL97* (Sheldrick, 2008 ▶); molecular graphics: *X-SEED* (Barbour, 2001 ▶); software used to prepare material for publication: *publCIF* (Westrip, 2010 ▶).

## Supplementary Material

Crystal structure: contains datablocks global, I. DOI: 10.1107/S1600536809055731/bt5161sup1.cif

Structure factors: contains datablocks I. DOI: 10.1107/S1600536809055731/bt5161Isup2.hkl

Additional supplementary materials:  crystallographic information; 3D view; checkCIF report

## Figures and Tables

**Fig. 1.**
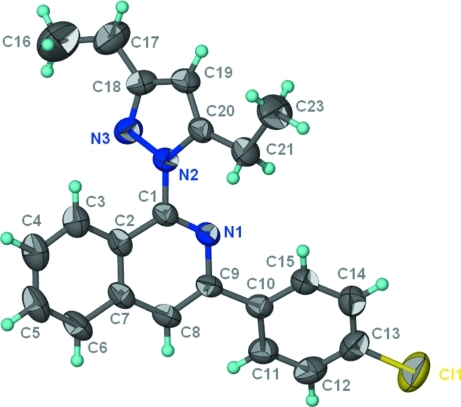
Anisotropic displacement ellipsoid plot (Barbour, 2001) of C22H20ClN3 at the 50% probability level; hydrogen atoms are drawn as spheres of arbitrary radius.

## supplementary crystallographic information

### Experimental

1-(3-(4-Chlorophenyl)isoquinolin-1-yl)hydrazine (2.69 g, 10mmol) and heptane-3,5-dione (1.28 g, 10 mmol) were dissolved in ethanol (30 ml). The solution was heated for 12 h under a nitrogen atmosphere. The reaction was quenched with water; the compound was extracted with ethyl acetate. The ethyl acetate phase was washed with water, dried, concentrated and purified by column chromatography to yield a white powder. Crystals were were obtained upon recrystallization from dichloromethane.

### Refinement

Hydrogen atoms were placed in calculated positions (C–H 0.93–0.97, O–H 0.82 Å) and were included in the refinement in the riding model approximation, with *U*(H) set to 1.2–1.5*U*(C).

### Crystal data

**Table:**

C_22_H_20_ClN_3_	*F*(000) = 760
*M**_r_* = 361.86	*D*_x_ = 1.230 Mg m^−^^3^
Monoclinic, *P*2_1_/*n*	Mo *K*α radiation, λ = 0.71073 Å
Hall symbol: -P 2yn	Cell parameters from 2329 reflections
*a* = 8.4484 (6) Å	θ = 2.6–20.1°
*b* = 15.0386 (12) Å	µ = 0.21 mm^−^^1^
*c* = 15.4894 (11) Å	*T* = 290 K
β = 96.763 (1)°	Block, colorless
*V* = 1954.3 (3) Å^3^	0.25 × 0.18 × 0.15 mm
*Z* = 4	

### Data collection

**Table:**

Bruker SMART area-detector diffractometer	3703 independent reflections
Radiation source: fine-focus sealed tube	2235 reflections with *I* > 2σ(*I*)
graphite	*R*_int_ = 0.040
φ and ω scans	θ_max_ = 25.7°, θ_min_ = 1.9°
Absorption correction: multi-scan (*SADABS*; Sheldrick, 1996)	*h* = −10→9
*T*_min_ = 0.951, *T*_max_ = 0.970	*k* = −18→18
14613 measured reflections	*l* = −18→18

### Refinement

**Table:**

Refinement on *F*^2^	Primary atom site location: structure-invariant direct methods
Least-squares matrix: full	Secondary atom site location: difference Fourier map
*R*[*F*^2^ > 2σ(*F*^2^)] = 0.062	Hydrogen site location: inferred from neighbouring sites
*wR*(*F*^2^) = 0.177	H-atom parameters constrained
*S* = 1.03	*w* = 1/[σ^2^(*F*_o_^2^) + (0.081*P*)^2^ + 0.4577*P*] where *P* = (*F*_o_^2^ + 2*F*_c_^2^)/3
3703 reflections	(Δ/σ)_max_ = 0.001
235 parameters	Δρ_max_ = 0.46 e Å^−^^3^
0 restraints	Δρ_min_ = −0.19 e Å^−^^3^

### Fractional atomic coordinates and isotropic or equivalent isotropic displacement parameters (Å^2^)

**Table:**

	*x*	*y*	*z*	*U*_iso_*/*U*_eq_	
Cl1	0.04502 (15)	0.45817 (9)	0.90130 (7)	0.1233 (5)	
N1	0.3419 (2)	0.36347 (13)	0.53000 (14)	0.0507 (5)	
N2	0.3322 (3)	0.30349 (14)	0.39224 (14)	0.0567 (6)	
N3	0.3354 (3)	0.34178 (16)	0.31188 (16)	0.0691 (7)	
C1	0.4218 (3)	0.34365 (16)	0.46595 (17)	0.0508 (6)	
C2	0.5882 (3)	0.35802 (16)	0.46517 (18)	0.0539 (7)	
C3	0.6777 (4)	0.3266 (2)	0.4002 (2)	0.0708 (8)	
H3	0.6272	0.2958	0.3526	0.085*	
C4	0.8377 (4)	0.3411 (2)	0.4069 (3)	0.0842 (10)	
H4	0.8964	0.3191	0.3645	0.101*	
C5	0.9138 (4)	0.3887 (2)	0.4766 (3)	0.0838 (10)	
H5	1.0228	0.3992	0.4797	0.101*	
C6	0.8312 (3)	0.4201 (2)	0.5405 (2)	0.0740 (9)	
H6	0.8841	0.4519	0.5867	0.089*	
C7	0.6650 (3)	0.40456 (16)	0.53719 (19)	0.0561 (7)	
C8	0.5763 (3)	0.42863 (17)	0.60411 (18)	0.0572 (7)	
H8	0.6246	0.4608	0.6513	0.069*	
C9	0.4196 (3)	0.40544 (16)	0.60104 (16)	0.0497 (6)	
C10	0.3237 (3)	0.42168 (17)	0.67334 (17)	0.0527 (7)	
C11	0.3598 (4)	0.48974 (19)	0.73263 (18)	0.0660 (8)	
H11	0.4439	0.5280	0.7257	0.079*	
C12	0.2736 (4)	0.5017 (2)	0.8014 (2)	0.0806 (9)	
H12	0.2991	0.5477	0.8408	0.097*	
C13	0.1495 (4)	0.4454 (2)	0.8118 (2)	0.0756 (9)	
C14	0.1079 (4)	0.3787 (2)	0.7535 (2)	0.0753 (9)	
H14	0.0224	0.3415	0.7603	0.090*	
C15	0.1948 (3)	0.3675 (2)	0.68431 (19)	0.0654 (8)	
H15	0.1664	0.3226	0.6442	0.078*	
C16	0.3239 (8)	0.3364 (4)	0.1205 (3)	0.170 (2)	
H16A	0.2865	0.3496	0.0609	0.254*	
H16B	0.3950	0.2865	0.1229	0.254*	
H16C	0.3792	0.3871	0.1468	0.254*	
C17	0.1949 (6)	0.3162 (3)	0.1649 (2)	0.1121 (14)	
H17A	0.1385	0.2663	0.1361	0.135*	
H17B	0.1227	0.3666	0.1599	0.135*	
C18	0.2325 (4)	0.2938 (2)	0.25986 (19)	0.0704 (8)	
C19	0.1668 (4)	0.2265 (2)	0.30509 (19)	0.0678 (8)	
H19	0.0936	0.1841	0.2820	0.081*	
C20	0.2294 (3)	0.23392 (18)	0.38943 (19)	0.0580 (7)	
C21	0.2030 (4)	0.1809 (2)	0.4678 (2)	0.0755 (9)	
H21A	0.3054	0.1626	0.4973	0.091*	
H21B	0.1518	0.2183	0.5074	0.091*	
C23	0.1027 (5)	0.1005 (3)	0.4464 (3)	0.1153 (15)	
H23A	0.0896	0.0689	0.4989	0.173*	
H23B	0.1538	0.0626	0.4082	0.173*	
H23C	0.0002	0.1183	0.4183	0.173*	

### Atomic displacement parameters (Å^2^)

**Table:**

	*U*^11^	*U*^22^	*U*^33^	*U*^12^	*U*^13^	*U*^23^
Cl1	0.1396 (10)	0.1493 (10)	0.0907 (8)	0.0002 (8)	0.0536 (7)	−0.0141 (7)
N1	0.0447 (12)	0.0527 (12)	0.0538 (13)	−0.0062 (10)	0.0020 (10)	−0.0004 (10)
N2	0.0556 (14)	0.0604 (14)	0.0539 (14)	−0.0113 (11)	0.0048 (11)	−0.0057 (11)
N3	0.0807 (18)	0.0707 (16)	0.0554 (15)	−0.0148 (13)	0.0055 (13)	0.0011 (12)
C1	0.0492 (16)	0.0455 (14)	0.0565 (17)	−0.0060 (11)	0.0009 (13)	0.0010 (12)
C2	0.0451 (15)	0.0481 (14)	0.0683 (18)	−0.0035 (12)	0.0062 (13)	0.0085 (13)
C3	0.063 (2)	0.0708 (19)	0.081 (2)	0.0019 (15)	0.0174 (17)	0.0037 (16)
C4	0.061 (2)	0.087 (2)	0.109 (3)	0.0079 (17)	0.027 (2)	0.010 (2)
C5	0.0443 (18)	0.088 (2)	0.120 (3)	−0.0014 (17)	0.012 (2)	0.027 (2)
C6	0.0460 (17)	0.077 (2)	0.095 (2)	−0.0096 (15)	−0.0072 (17)	0.0142 (18)
C7	0.0455 (15)	0.0492 (15)	0.0711 (18)	−0.0055 (12)	−0.0042 (14)	0.0141 (13)
C8	0.0540 (17)	0.0527 (15)	0.0609 (17)	−0.0079 (13)	−0.0097 (14)	0.0028 (13)
C9	0.0480 (16)	0.0459 (14)	0.0523 (16)	−0.0046 (12)	−0.0064 (12)	0.0045 (12)
C10	0.0516 (16)	0.0515 (15)	0.0523 (15)	0.0029 (12)	−0.0049 (12)	0.0064 (12)
C11	0.074 (2)	0.0614 (17)	0.0616 (18)	−0.0055 (15)	0.0018 (16)	−0.0036 (15)
C12	0.098 (3)	0.080 (2)	0.062 (2)	−0.0021 (19)	0.0049 (18)	−0.0152 (17)
C13	0.084 (2)	0.087 (2)	0.0566 (19)	0.0108 (19)	0.0129 (16)	0.0040 (17)
C14	0.070 (2)	0.084 (2)	0.073 (2)	−0.0059 (17)	0.0134 (17)	0.0072 (18)
C15	0.0614 (18)	0.0712 (19)	0.0627 (18)	−0.0079 (15)	0.0035 (15)	−0.0034 (14)
C16	0.183 (6)	0.225 (7)	0.097 (4)	−0.021 (5)	0.000 (4)	0.037 (4)
C17	0.162 (4)	0.105 (3)	0.071 (3)	−0.028 (3)	0.020 (3)	−0.007 (2)
C18	0.078 (2)	0.078 (2)	0.0541 (18)	−0.0048 (17)	0.0019 (16)	−0.0130 (16)
C19	0.0670 (19)	0.0709 (19)	0.065 (2)	−0.0160 (15)	0.0042 (15)	−0.0193 (16)
C20	0.0536 (16)	0.0571 (16)	0.0639 (18)	−0.0119 (13)	0.0100 (13)	−0.0113 (14)
C21	0.083 (2)	0.0689 (19)	0.076 (2)	−0.0276 (16)	0.0127 (17)	−0.0040 (16)
C23	0.142 (4)	0.096 (3)	0.109 (3)	−0.060 (3)	0.015 (3)	0.001 (2)

### Geometric parameters (Å, °)

**Table:**

Cl1—C13	1.739 (3)	C11—H11	0.9300
N1—C1	1.299 (3)	C12—C13	1.372 (5)
N1—C9	1.368 (3)	C12—H12	0.9300
N2—C20	1.357 (3)	C13—C14	1.368 (4)
N2—N3	1.375 (3)	C14—C15	1.378 (4)
N2—C1	1.428 (3)	C14—H14	0.9300
N3—C18	1.327 (4)	C15—H15	0.9300
C1—C2	1.424 (4)	C16—C17	1.390 (6)
C2—C7	1.409 (4)	C16—H16A	0.9600
C2—C3	1.410 (4)	C16—H16B	0.9600
C3—C4	1.361 (4)	C16—H16C	0.9600
C3—H3	0.9300	C17—C18	1.505 (5)
C4—C5	1.389 (5)	C17—H17A	0.9700
C4—H4	0.9300	C17—H17B	0.9700
C5—C6	1.361 (4)	C18—C19	1.384 (4)
C5—H5	0.9300	C19—C20	1.355 (4)
C6—C7	1.418 (4)	C19—H19	0.9300
C6—H6	0.9300	C20—C21	1.491 (4)
C7—C8	1.396 (4)	C21—C23	1.491 (4)
C8—C9	1.364 (4)	C21—H21A	0.9700
C8—H8	0.9300	C21—H21B	0.9700
C9—C10	1.478 (4)	C23—H23A	0.9600
C10—C15	1.387 (4)	C23—H23B	0.9600
C10—C11	1.385 (4)	C23—H23C	0.9600
C11—C12	1.372 (4)		
			
C1—N1—C9	118.5 (2)	C14—C13—Cl1	119.6 (3)
C20—N2—N3	112.0 (2)	C12—C13—Cl1	119.5 (3)
C20—N2—C1	129.0 (2)	C13—C14—C15	119.0 (3)
N3—N2—C1	118.8 (2)	C13—C14—H14	120.5
C18—N3—N2	103.9 (2)	C15—C14—H14	120.5
N1—C1—C2	125.1 (2)	C14—C15—C10	121.4 (3)
N1—C1—N2	115.7 (2)	C14—C15—H15	119.3
C2—C1—N2	119.3 (2)	C10—C15—H15	119.3
C7—C2—C3	119.8 (3)	C17—C16—H16A	109.5
C7—C2—C1	115.6 (3)	C17—C16—H16B	109.5
C3—C2—C1	124.6 (3)	H16A—C16—H16B	109.5
C4—C3—C2	120.2 (3)	C17—C16—H16C	109.5
C4—C3—H3	119.9	H16A—C16—H16C	109.5
C2—C3—H3	119.9	H16B—C16—H16C	109.5
C3—C4—C5	120.4 (3)	C16—C17—C18	116.5 (4)
C3—C4—H4	119.8	C16—C17—H17A	108.2
C5—C4—H4	119.8	C18—C17—H17A	108.2
C6—C5—C4	121.0 (3)	C16—C17—H17B	108.2
C6—C5—H5	119.5	C18—C17—H17B	108.2
C4—C5—H5	119.5	H17A—C17—H17B	107.3
C5—C6—C7	120.4 (3)	N3—C18—C19	111.3 (3)
C5—C6—H6	119.8	N3—C18—C17	121.3 (3)
C7—C6—H6	119.8	C19—C18—C17	127.4 (3)
C8—C7—C2	118.6 (2)	C20—C19—C18	107.1 (3)
C8—C7—C6	123.1 (3)	C20—C19—H19	126.5
C2—C7—C6	118.2 (3)	C18—C19—H19	126.5
C9—C8—C7	120.7 (3)	C19—C20—N2	105.7 (3)
C9—C8—H8	119.6	C19—C20—C21	131.5 (3)
C7—C8—H8	119.6	N2—C20—C21	122.9 (2)
C8—C9—N1	121.2 (3)	C23—C21—C20	112.8 (3)
C8—C9—C10	123.2 (2)	C23—C21—H21A	109.0
N1—C9—C10	115.6 (2)	C20—C21—H21A	109.0
C15—C10—C11	117.9 (3)	C23—C21—H21B	109.0
C15—C10—C9	120.3 (2)	C20—C21—H21B	109.0
C11—C10—C9	121.8 (3)	H21A—C21—H21B	107.8
C12—C11—C10	121.1 (3)	C21—C23—H23A	109.5
C12—C11—H11	119.5	C21—C23—H23B	109.5
C10—C11—H11	119.5	H23A—C23—H23B	109.5
C11—C12—C13	119.6 (3)	C21—C23—H23C	109.5
C11—C12—H12	120.2	H23A—C23—H23C	109.5
C13—C12—H12	120.2	H23B—C23—H23C	109.5
C14—C13—C12	121.0 (3)		
			
C20—N2—N3—C18	−0.2 (3)	C8—C9—C10—C15	−152.8 (3)
C1—N2—N3—C18	−175.2 (2)	N1—C9—C10—C15	26.0 (3)
C9—N1—C1—C2	3.5 (4)	C8—C9—C10—C11	26.3 (4)
C9—N1—C1—N2	−177.3 (2)	N1—C9—C10—C11	−154.9 (2)
C20—N2—C1—N1	−49.0 (4)	C15—C10—C11—C12	1.8 (4)
N3—N2—C1—N1	125.1 (3)	C9—C10—C11—C12	−177.3 (3)
C20—N2—C1—C2	130.3 (3)	C10—C11—C12—C13	0.0 (5)
N3—N2—C1—C2	−55.7 (3)	C11—C12—C13—C14	−1.5 (5)
N1—C1—C2—C7	−5.9 (4)	C11—C12—C13—Cl1	177.7 (2)
N2—C1—C2—C7	174.9 (2)	C12—C13—C14—C15	1.3 (5)
N1—C1—C2—C3	171.8 (3)	Cl1—C13—C14—C15	−178.0 (2)
N2—C1—C2—C3	−7.3 (4)	C13—C14—C15—C10	0.6 (5)
C7—C2—C3—C4	0.0 (4)	C11—C10—C15—C14	−2.1 (4)
C1—C2—C3—C4	−177.7 (3)	C9—C10—C15—C14	177.0 (3)
C2—C3—C4—C5	−1.4 (5)	N2—N3—C18—C19	−0.6 (3)
C3—C4—C5—C6	1.4 (5)	N2—N3—C18—C17	177.6 (3)
C4—C5—C6—C7	0.2 (5)	C16—C17—C18—N3	44.6 (6)
C3—C2—C7—C8	−174.8 (2)	C16—C17—C18—C19	−137.5 (5)
C1—C2—C7—C8	3.0 (3)	N3—C18—C19—C20	1.2 (4)
C3—C2—C7—C6	1.5 (4)	C17—C18—C19—C20	−176.8 (3)
C1—C2—C7—C6	179.3 (2)	C18—C19—C20—N2	−1.2 (3)
C5—C6—C7—C8	174.6 (3)	C18—C19—C20—C21	179.6 (3)
C5—C6—C7—C2	−1.6 (4)	N3—N2—C20—C19	0.9 (3)
C2—C7—C8—C9	1.8 (4)	C1—N2—C20—C19	175.3 (3)
C6—C7—C8—C9	−174.3 (2)	N3—N2—C20—C21	−179.8 (3)
C7—C8—C9—N1	−4.5 (4)	C1—N2—C20—C21	−5.4 (4)
C7—C8—C9—C10	174.2 (2)	C19—C20—C21—C23	7.2 (5)
C1—N1—C9—C8	1.9 (3)	N2—C20—C21—C23	−171.8 (3)
C1—N1—C9—C10	−176.9 (2)		
